# Supplementary material for: Elevated Na+/K+ Ratio in Alzheimer’s Disease: A Potential Biomarker for Braak Stage
Source: Int J Mol Sci. 2026 Jan 27;27(3):1247. doi: 10.3390/ijms27031247 (PMC12898237; doi:10.3390/ijms27031247)
Supplement: Supplementary file 1 [file ijms-27-01247-s001.zip › ijms-3859782-supplementary.pdf]

Supplemental Data:

Elevated Na^+/K^+ Ratio in Alzheimer's Disease: A Potential Biomarker for Braak Stage

Yuma Mizuno [1], Shiyue Pan [2,3], Tong Zhou [1], Patrick G. Kehoe [4] and Yumei Feng Earley [2,3#]

1. Department of Physiology and Cell Biology, University of Nevada, Reno, NV, USA
2. Department of Medicine, University of Rochester Medical Center, Rochester, NY, USA
3. Department of Pharmacology & Physiology, University of Rochester Medical Center, Rochester, NY, USA
4. Cerebrovascular and Dementia Research Group, Translational Health Sciences, Bristol Medical School, University of Bristol, Bristol, UK

[#]Correspondence to:
Yumei Feng Earley MD, PhD, FAHA
Professor of Medicine, Pharmacology & Physiology
University of Rochester Medical Center
601 Elmwood Avenue,
Rochester, NY 14642, USA
E-mail: Yumei_Earley@URMC.Rochester.edu

Figure S1

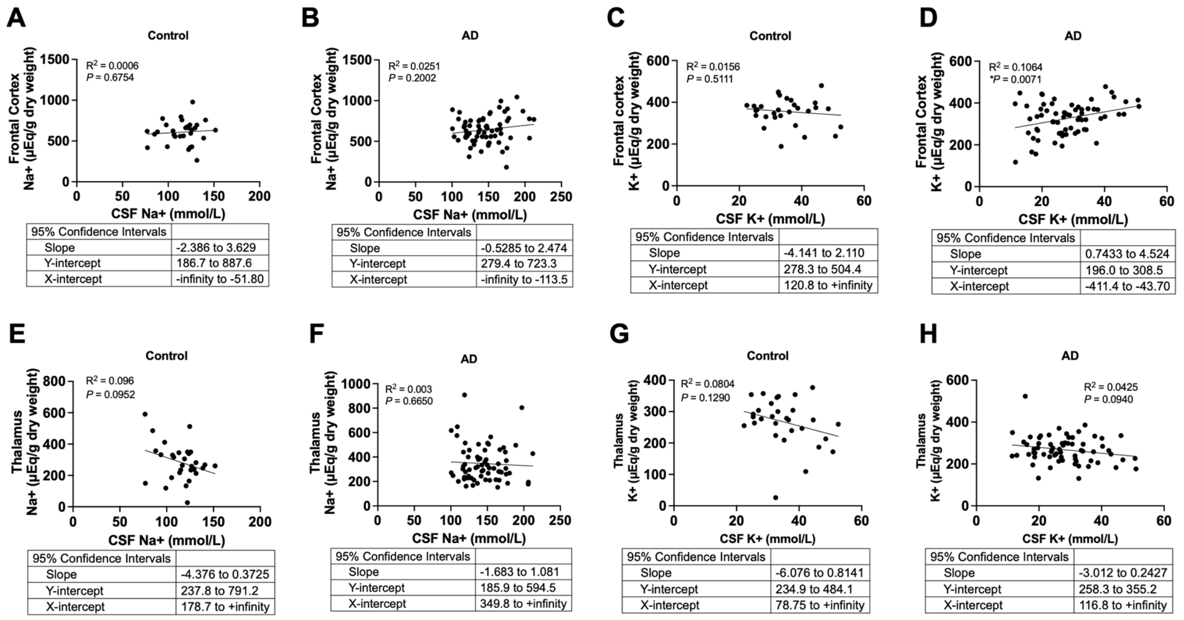

A

95% Confidence Intervals	
Slope	-2.386 to 3.629
Y-intercept	186.7 to 887.6
X-intercept	-infinity to -51.80

B

95% Confidence Intervals	
Slope	-0.5285 to 2.474
Y-intercept	279.4 to 723.3
X-intercept	-infinity to -113.5

C

95% Confidence Intervals	
Slope	-4.141 to 2.110
Y-intercept	278.3 to 504.4
X-intercept	120.8 to +infinity

D

95% Confidence Intervals	
Slope	0.7433 to 4.524
Y-intercept	196.0 to 308.5
X-intercept	-411.4 to -43.70

E

95% Confidence Intervals	
Slope	-4.376 to 0.3725
Y-intercept	237.8 to 791.2
X-intercept	178.7 to +infinity

F

95% Confidence Intervals	
Slope	-1.683 to 1.081
Y-intercept	185.9 to 594.5
X-intercept	349.8 to +infinity

G

95% Confidence Intervals	
Slope	-6.076 to 0.8141
Y-intercept	234.9 to 484.1
X-intercept	78.75 to +infinity

H

95% Confidence Intervals	
Slope	-3.012 to 0.2427
Y-intercept	258.3 to 355.2
X-intercept	116.8 to +infinity

Figure S1. Relationship between Cerebrospinal fluid (CSF) and brain tissue sodium (Na^+) and potassium (K^+) concentrations in control and Alzheimer's disease (AD) subjects. Linear regression analyses were performed to assess correlations between ion concentrations in the frontal cortex and cerebrospinal fluid (CSF) in control and Alzheimer's disease (AD) subjects. (A, B) Frontal cortex versus CSF [Na^+] in control and AD groups. (C, D) Frontal cortex versus CSF [K^+] in control and AD groups. (E, F) Thalamic versus CSF [Na^+] in control and AD groups. (G, H) Thalamic versus CSF [K^+] in control and AD groups. Simple Linear Regression, N = 97. Two-tailed, and differences were considered statistically significant at *P*-values < 0.05.

Figure S2

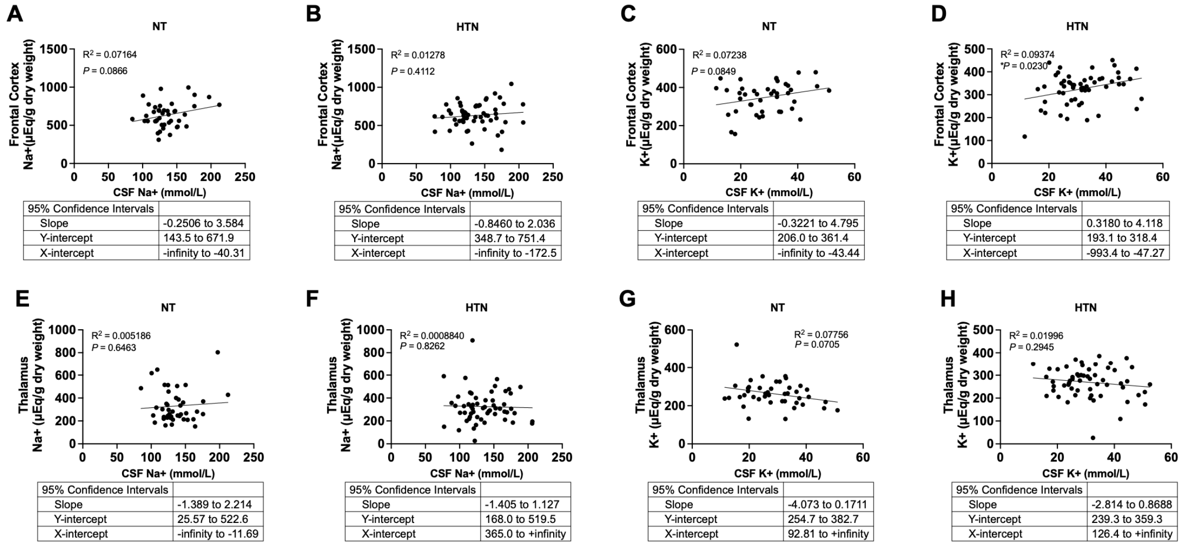

Figure S2. Relationship between Cerebrospinal fluid (CSF) and brain tissue sodium (Na^+) and potassium (K^+) concentrations in normotensive (NT) and hypertensive (HT) subjects. Linear regression analyses were performed to assess correlations between ion concentrations in the frontal cortex and cerebrospinal fluid (CSF) in NT and HTN subjects. (A, B) Frontal cortex versus CSF [Na^+] in NT and HTN subjects. (C, D) Frontal cortex versus CSF [K^+] in NT and HTN subjects. (E, F) Thalamic versus CSF [Na^+] in NT and HTN subjects. (G, H) Thalamic versus CSF [K^+] in NT and HTN subjects. Simple Linear Regression, N = 97. Two-tailed, and differences were considered statistically significant at *P*-values < 0.05.
